# Comparative analysis of growth performance and rumen microbiota in Hu sheep and its three-way crossbred offspring

**DOI:** 10.3389/fmicb.2026.1773833

**Published:** 2026-05-04

**Authors:** Haina Shi, Zhenfei Xu, Zhenghan Chen, Shujun Shi, Chune Niu, Xuejiao An, Qiao Li, Zhiguang Geng, Jinxia Zhang, Rui Zhang, Yaojing Yue

**Affiliations:** 1 Country Sheep Breeding Engineering Technology Research Center, Chinese Academy of Agricultural Sciences, Lanzhou, Gansu, China; 2 Lanzhou Institute of Husbandry and Pharmaceutical Sciences, Chinese Academy of Agricultural Sciences, Lanzhou, Gansu, China; 3 Qingyang Research Institute of Agricultural Sciences, Qingyang, Gansu, China

**Keywords:** growth performance, Hu sheep, rumen microbiota, three-way crossbred, volatile fatty acids

## Abstract

This study compared the growth performance, rumen fermentation patterns, and microbial community structure between three-way crossbred (Poll Dorset × Southdown × Hu, TNH) and purebred Hu (HH) sheep. A total of 75 male lambs (HH: *n* = 35; TNH: *n* = 40) with comparable birth dates were selected. After a 56-day suckling period, all lambs underwent an 84-day standardized fattening protocol with a uniform diet. Growth indicators were monitored, and rumen fluid was collected using an oral ruminal sampler for the determination of volatile fatty acids (VFAs) and 16S rRNA sequencing. TNH sheep demonstrated significantly superior growth performance, including higher body weight (BW), average daily gain (ADG) and Kleiber ratio (KR). TNH sheep exhibited an acetate-type pattern with higher acetate, butyrate, total VFAs, and acetate/propionate ratio (A/P), whereas HH sheep showed a propionate-type fermentation pattern with higher branched-chain VFAs. Bacteroidetes and Firmicutes were the dominant core microbiota in both the TNH and HH groups. However, the higher species richness and diversity in the TNH sheep was observed, and harbored a distinct community structure featuring *Prevotella* as the predominant genus. *Shuttleworthia*, *Olsenella*, and *Syntrophococcus* were unique to HH sheep, whereas *Prevotellaceae_UCG_003* was specifically enriched in the TNH sheep. Metabolic pathways, carbon metabolism, and glycolysis/gluconeogenesis were predominantly enriched in TNH sheep, whereas biosynthesis of amino acids and ABC transporters were more abundant in HH sheep. These findings demonstrate that crossbreeding can optimize host metabolism and production performance by modulating rumen VFA dynamics and microbiota structure.

## 1 Introduction

Digestive tract microorganisms can regulate lipid metabolism, energy balance, and immune response in animals and are thus called the “second genome” (Kostka, 2025). For ruminants, the rumen is a crucial part of the digestive tract and the natural fermentation tank with the strongest ability to degrade fibrous substances. Approximately 40–80% of the dry matter is digested in the rumen, and the digestibility of crude fiber can reach 90% (Jung and Allen, 1995). The rumen is colonized by thousands of microorganisms, which, through long-term environmental adaptation, undergo synergistic coevolution with the host to ensure efficient nutrient digestion and absorption (Zhou et al., 2024). Within this symbiotic relationship, the host provides a suitable internal environment for the microbes, while the microbes metabolize dietary components to produce volatile fatty acids (VFAs) for host utilization. Importantly, microbial fermentation supplies nutrients, such as VFAs, that can meet up to 70% of the host energy requirements. VFAs thereby function as a central bridge in host-microbiota crosstalk, directly and indirectly modulating host metabolic pathways, intestinal immune responses, and gut microbial composition (Wang et al., 2023; van der Hee and Wells, 2021). Several studies have reported a significant relationship between rumen microorganisms and host growth performance traits, such as body weight, feed conversion rate, and meat production (Wang et al., 2022; Zhao et al., 2025; Zhou and Shen, 2025; Li et al., 2025). However, the microbial community varies among breeds and is influenced by host genetics, sex, environment, diet, age, and geographic range (Wang et al., 2023; Li et al., 2022; Liu et al., 2021; Furman et al., 2020; Li et al., 2016; Wei et al., 2025).

As an important breeding strategy, crossbreeding improves sheep production performance by enhancing offspring growth, increasing daily gain, and decreasing the feed conversion ratio (FCR). Nevertheless, the synergistic mechanisms between crossbreeding and the rumen microbiome remain to be fully elucidated. The Hu sheep is a prolific breed known for its high reproductive efficiency, good maternal traits, and non-seasonal estrous cycles. The stable heritability of the multi-lambing trait makes the Hu sheep a crucial genetic resource for sheep breeding programs (Shi et al., 2024; Zhang et al., 2025). Therefore, Hu sheep are typically selected as the female parent in crossbreeding strategies to develop new strains and varieties with superior reproductive performance (China National Commission of Animal Genetic Esources, 2011). The Southdown sheep is a short-haired meat sheep breed, the oldest native breed in the UK, renowned for its gentle temperament, superior meat quality, and desirable carcass characteristics (Liu et al., 2023; Kong et al., 2023). It was once preeminent in the production of high-quality lambs and has been introduced to many countries to serve as a sire breed for fat lamb production (Lu et al., 2022). The Poll Dorset is known for its fast growth rate, early maturity, and superior meat performance (Miao et al., 2023). In China, Southdown and Poll Dorset sheep are considered valuable paternal genetic resources for crossbreeding programs aimed at improving production performance of local sheep. The utilization of Poll Dorset rams in crossbreeding with local sheep results in notable heterosis, mitigating growth retardation in indigenous breeds. Research demonstrates that crossing Hu sheep ewes with meat-type sheep breeds leads to significant improvements in growth performance, meat production traits, and carcass characteristics in the offspring (Zhang et al., 2025; Lu et al., 2022). Recently, most studies on Hu sheep have focused on improving growth performance, slaughter performance, and meat quality through purebred selection and two-way crossbreeding (Liu et al., 2023; Wang et al., 2024; Zhang et al., 2024; Zhang et al., 2023; Hong et al., 2023; Xu et al., 2023; Zhan et al., 2026). However, the use of Hu sheep in three-way crossbreeding systems with terminal paternal breeds remains limited.

In this study, Poll Dorset rams were used as terminal sires to cross with Southdown × Hu hybrid ewes, producing a three-way cross (Poll Dorset × Southdown × Hu, designated as TNH). Under identical rearing conditions, the TNH crossbred lambs were then compared with purebred Hu (HH) lambs in terms of growth performance, body size, rumen fermentation parameters, and microbial community structure. This study aims to provide new insights into enhancing offspring growth performance through crossbreeding.

## 2 Materials and methods

### 2.1 Animals and diets

This study was conducted at the Qinghuan Mutton Sheep Breeding Company in Huanxian County, Gansu, China. The experimental protocol was approved by the Institutional Animal Care and Use Committee of the Lanzhou Institute of Husbandry and Pharmaceutical Sciences, Chinese Academy of Agricultural Sciences (Approval No. 2024-14).

A total of 75 healthy male lambs of similar birth dates were selected, comprising 35 purebred HH (Hu × Hu, HH) and 40 three-way crossbred (Poll Dorset × Southdown × Hu, TNH) individuals. At 56 days of age, all lambs were weaned and subsequently subjected to an 84-day standardized fattening period, during which they received a uniform mixed diet (Table 1). Throughout the experiment, all animals had ad libitum access to feed and water, and all management practices (feeding and housing) were standardized.

**Table 1:** Ingredient composition and nutrient levels of experimental diets (dry matter basis, %).

Ingredients	Content	Nutrient level	Content
Oat hay	8.57	Dry matter	69.37
Leymus chinensis	16.76	Crude protein	13.81
Corn Silage	13.53	Acid detergent fiber	15.87
Corn Grain Whole	32.65	Neutral detergent fiber	28.62
Lamb9303	28.49	Fat	3.39
Totals	100.00	Starch	23.23
		Calcium	0.56
		Phosphorus	0.31

Lamb9303: mainly composed of soybean oil, soybean meal, cottonseed meal, rapeseed meal, DDGS, calcium carbonate, calcium nitrogen phosphate, sodium chloride, a variety of trace elements and vitamins; its crude protein content ≥ 32.0%, crude ash ≤ 21.0%, calcium is1.4–5.0%, total phosphorus ≥ 0.60%, crude fiber ≤ 20.0%, lysine ≥ 1.0%, sodium chloride is 1.5–5.0%.

### 2.2 Growth performance measurement

All lambs were weighed at birth (day 0, BW_0 d_), weaning (day 56, BW_56 d_), and at the end of the trial (day 140, BW_140 d_) using a calibrated electronic scale. Average daily gain (ADG) was calculated. Additionally, at 140 days of age, body size indices were recorded using a flexible tape, including body height (BH), body length (BL), chest circumference (CC), and tube circumference (TC). All measurements were standardized and performed in the morning before feeding. Then, the Kleiber ratio was calculated as KR = $\frac{\mathrm{ADG}}{{\mathrm{BW}}^{0.75}}$ × 100%.

### 2.3 Rumen fermentation parameters measurement

At 140 days of age, 50 mL of rumen fluid was collected from each sheep using a gastric tube rumen sampler prior to morning feeding. The first portion of the sample was discarded to avoid saliva contamination, the remainder was filtered through four layers of cheesecloth, and the resulting filtrate was aliquoted into 5 mL cryogenic vials. The vials were then snap-frozen in liquid nitrogen, transported to the laboratory, and stored at −80 °C until analysis. Acetate (AA), propionate (AP), isobutyrate (IBA), butyrate (BA), isovalerate (IVA), and valerate (VA) were determined by gas chromatograph (QP2010-Ultra, Shimadzu, Japan) according to Liu et al. (2020). Subsequently, the concentrations of total volatile fatty acids (TVFAs), the acetate/propionate ratio (A/P), and the molar proportions of individual VFAs, including acetate (AAR), propionate (PAR), isobutyrate (IBAR), butyrate (BAR), isovalerate (IVAR), and valerate (VAR), were calculated.

### 2.4 DNA extraction and 16S rRNA sequencing

To extract DNA from rumen fluid, 1.5 mL of rumen fluid was centrifuged at 10,000 × g for 10 min at 4 °C. The supernatant was discarded, and the microbial pellet was used for DNA extraction using the E. Z. N. A.^®^ Soil DNA Kit (Omega Bio-tek, Norcross, GA, United States) following the manufacturer’s protocol. DNA purity and concentration were measured with a NanoDrop2000 spectrophotometer (Thermo Scientific, United States), and DNA integrity was assessed by 1.0% agarose gel electrophoresis. The hypervariable region V3-V4 of the bacterial 16S rRNA gene was amplified with forward primer 338F (5′-ACTCCTACGGGAGGCAGCAG-3′) and reverse primer 806R (5′-GGACTACHVGGGTWTCTAAT-3′) (Wang et al., 2024) using a T100 Thermal Cycler PCR (BIO-RAD, United States). The PCR reaction mixture (20 μL) contained 4 μL of 5 × Fast Pfu buffer, 2 μL of 2.5 mM dNTPs, 0.8 μL of each primer (5 μM), 0.4 μL of Fast Pfu polymerase, 10 ng of template DNA, and ddH_2_O to make up the volume. PCR amplification cycling conditions were as follows: initial denaturation at 95 °C for 3 min; followed by 27 cycles of denaturing at 95 °C for 30 s, annealing at 55 °C for 30 s and extension at 72 °C for 45 s; and single extension at 72 °C for 10 min, and a hold at 4 °C. The PCR product was extracted from a 2% agarose gel and purified using the PCR Clean-Up Kit (YuHua, Shanghai, China) according to the manufacturer’s instructions, and quantified using a Qubit 4.0 fluorometer (Thermo Fisher Scientific, United States). Purified amplicons were pooled in equimolar concentrations and subjected to paired-end sequencing on an Illumina NextSeq 2000 platform (Illumina, San Diego, CA, United States) using the manufacturer’s recommended reagents.

Quality filtering of the demultiplexed sequences was performed with fastp (v0.19.6) (Chen et al., 2018; Edgar, 2013), and the filtered sequences were then merged using FLASH (version 1.2.11) (Magoč and Salzberg, 2011). Subsequently, high-quality sequences were denoised using the DADA2 plugin in QIIME2 (version 2020.2) (Wang et al., 2007) with recommended parameters.

This denoising process, which achieves single-nucleotide resolution by modeling sample-specific error profiles, produces amplicon sequence variants (ASVs). To minimize the impact of uneven sequencing depth on diversity analyses, the data were rarefied to 20,000 sequences per sample, yielding an average Good’s coverage of 97.00%. Taxonomic assignment of ASVs was performed using the Naive Bayes consensus taxonomy classifier implemented in QIIME2 and the SILVA (v138) 16S rRNA database. Microbial functions were predicted using PICRUSt2 (Douglas et al., 2020) based on ASV representative sequences.

Bioinformatics analysis of the rumen microbiota was performed using the Majorbio Cloud platform (Han et al., 2024). Based on the ASV information, rarefaction curves and alpha diversity indices were calculated using Mothur v1.30.2 (Schloss et al., 2009). The similarity among microbial communities in different samples was determined using principal coordinate analysis (PCoA) based on Bray–Curtis dissimilarity with the Vegan v2.5–3 package. PERMANOVA was performed using the Vegan v2.5–3 package to determine the proportion of variation explained by treatment and its statistical significance. Linear discriminant analysis (LDA) effect size (LEfSe) (Segata et al., 2011) was performed to identify bacterial taxa with significant abundance differences among groups from phylum to genus level (LDA score > 4, *p* < 0.05).

### 2.5 Statistical analysis

Body size indices, KR, and VFAs were analyzed using Student’s *t*-test in SPSS statistics software (v23.0, IBM, Chicago, United States). The data were presented as means ± SEM. A *p* < 0.05 was considered statistically significant, *p* < 0.01 was defined highly significant, and values between 0.05 and 0.10 were interpreted as suggesting a trend.

## 3 Results

### 3.1 Crossbreeding improved the growth performance in HH and TNH sheep

Growth performance of HH and TNH sheep was determined (Table 2). The BW_0 d_ and BW_140 d_ in the TNH group were significantly higher than those in the HH group (*p* < 0.001), while weaning weight showed an increasing trend compared to the HH group (*p* = 0.099). Average daily gain (ADG) during all periods (0–56 d, 56–140 d, and 0–140 d) was significantly higher in the TNH group than in the HH group (*p* < 0.001). The Kleiber ratio, which assesses growth efficiency and feed utilization capacity, was significantly higher in the TNH group than that in the HH group (*p* < 0.001). For body size measurements, chest circumference (CC) and tube circumference (TC) at 140 days of age were significantly higher in the TNH group than those in the HH group (*p* < 0.001). However, no significant differences were observed in body height (BH) or body length (BL) between the two groups.

**Table 2:** Comparative analysis of growth performance in HH and TNH Sheep.

Measurements	HH	TNH	*p*-value
BW			
BW_0 d_, kg	2.86 ± 0.57^b^	3.53 ± 0.69^a^	0.001
BW_56 d_, kg	15.36 ± 5.85	17.82 ± 1.75	0.099
BW_140 d_, kg	33.13 ± 3.98^b^	37.42 ± 5.16^a^	<0.001
ADG			
ADG_0-56 d_, g	208.38 ± 23.36^b^	238.10 ± 30.56^a^	<0.001
ADG_56-140 d_, g	177.74 ± 19.50^b^	196.07 ± 25.29^a^	<0.001
ADG_0-140 d_, g	192.00 ± 21.73^b^	230.10 ± 52.52^a^	<0.001
KR	1.39 ± 0.08^b^	1.53 ± 0.21^a^	<0.001
Body size			
BH, cm	64.69 ± 4.83	64.80 ± 3.09	0.909
BL, cm	66.21 ± 4.59	67.54 ± 3.93	0.179
CC, cm	76.13 ± 6.21^b^	86.12 ± 9.79^a^	<0.001
TC, cm	8.54 ± 0.89^b^	9.87 ± 0.82^a^	<0.001

BW, body weight; BW_0 d_, birth weight (day 0); BW_56 d_, weaned weight (day 56); BW_140 d_, body weight (day 140); BH, body height; BL, body length; CC, chest circumference; TC, tube circumference; ADG, average daily weight gain; ADG_0-56 d_, average daily weight gain from birth to weaning; ADG_56-140 d_, average daily weight gain from weaning to the end of fattening; ADG_0-140 d_, average daily weight gain from birth to the end of fattening; KR, Kleiber ratio. In the same row, values with no letter superscripts mean no significant difference (*p* > 0.05), while with small letter superscripts mean significant difference (*p* < 0.05).

A comprehensive correlation analysis demonstrated consistently positive relationships among growth indicators in both HH and TNH sheep (Table 3). BL was highly significantly correlated with BW_140 d_ and BH (*p* < 0.01), and TC was highly significantly correlated with BH, BL, and CC (*p* < 0.01). The maximum correlation coefficients were observed between BL and BH, reaching 0.592 in TNH sheep and 0.874 in HH sheep. CC was significantly correlated with BW_140 d_, BH, and BL in both TNH (*p* < 0.05) and HH (*p* < 0.01) sheep. Notably, in HH sheep, ADG_0-140 d_ showed extremely significant correlations with BW_140 d_, BH, BL, and CC (*p* < 0.01). Additionally, a significant correlation was found between BW_140 d_ and BH (*p* < 0.05). In contrast, ADG_0-140 d_ in TNH sheep was only highly significantly correlated with BW_140 d_ (*p* < 0.01).

**Table 3:** Correlation analysis of body weight and body size indices in HH and TNH sheep.

Item	ADG_0-140 d_	BW_140 d_	BH	BL	CC	TC
ADG_0-140 d_	1	0.483**	0.102	0.171	0.158	0.091
BW_140 d_	0.874**	1	0.306	0.463**	0.340*	0.287
BH	0.545**	0.425*	1	0.592**	0.396*	0.425**
BL	0.581**	0.473**	0.874**	1	0.340*	0.551**
CC	0.520**	0.510**	0.785**	0.777**	1	0.486**
TC	0.315	0.244	0.678**	0.701**	0.774**	1

The lower left corner is HH, the upper right corner is TNH. *Indicates significant difference (*p* < 0.01), **Indicates extremely significant difference (*p* < 0.01).

### 3.2 Crossbreeding altered rumen fermentation parameters in HH and TNH sheep

Herein, the ruminal volatile fatty acids of lambs were evaluated (Table 4). The concentrations of AA, BA, and TVFAs, as well as A/P, were significantly higher in the TNH group than those in the HH group (*p* < 0.05). However, the concentrations of IBA, VA, and IVA were significantly lower (*p* < 0.05). Moreover, the AAR in the TNH group was significantly higher than that in the HH group (*p* < 0.01), while the PAR, IBAR, VAR, and IVAR were significantly lower than those in the HH group (*p* < 0.01).

**Table 4:** Comparative analysis of rumen volatile fatty acids concentrations in HH and TNH sheep.

Items		HH	TNH	*p-*value
Molar concentration (mmol/kg)	Acetic acid (AA)	18.17 ± 8.20	30.94 ± 6.93^**^	<0.001
	Propionic acid (PA)	9.65 ± 5.86	8.03 ± 1.85	0.124
	Butyric acid (BA)	3.04 ± 1.55	3.72 ± 1.10^*^	0.036
	Isobutyric acid (IBA)	0.92 ± 0.27^**^	0.76 ± 0.11	0.003
	Valeric acid (VA)	1.07 ± 0.61^**^	0.37 ± 0.09	<0.001
	Isovaleric acid (IVA)	1.62 ± 0.55^**^	1.27 ± 0.21	0.001
	Total VFAs	36.64 ± 15.48	45.12 ± 9.70^**^	0.001
	A/P	2.04 ± 0.47	3.88 ± 0.37^**^	<0.001
Molar proportion	Acetic acid (AAR)	52.30 ± 4.22	68.45 ± 2.25^**^	<0.001
	Propionic acid (PAR)	26.64 ± 5.03^**^	17.77 ± 1.29	<0.001
	Butyric acid (BAR)	8.70 ± 1.67	8.20 ± 1.62	0.194
	Isobutyric acid (IBAR)	3.17 ± 1.53^**^	1.75 ± 0.38	<0.001
	Valeric acid (VAR)	3.14 ± 0.89^**^	0.83 ± 0.13	<0.001
	Isovaleric acid (IVAR)	5.53 ± 2.62^**^	2.91 ± 0.62	<0.001

### 3.3 Composition of rumen microbiota in HH and TNH sheep

**Figure 1:**
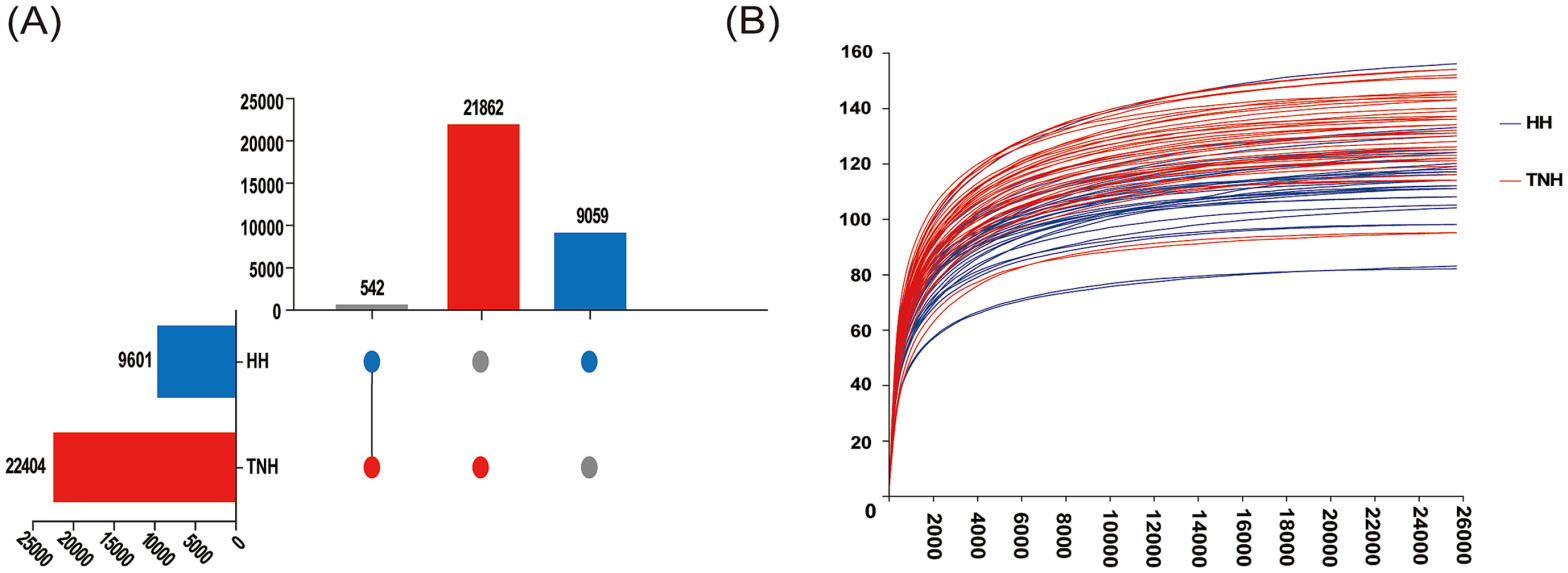
Characteristics of microbial composition and sequencing data assessment in TNH and HH sheep. **(A)** UpsetR figure of rumen ASVs. **(B)** Rarefaction curves.

16S rRNA sequencing was performed on rumen fluid samples from 35 HH and 40 TNH sheep. After quality filtering, denoising, and chimera removal, a total of 4,531,140 optimized sequences were obtained, with an average sequence length of 417 bp. From these sequences, 31,463 ASVs were identified, which were classified into 24 phyla, 50 classes, 109 orders, 190 families, and 412 genera. Group-specific analysis showed that TNH sheep harbored 22,404 unique ASVs, HH sheep harbored 9,601 unique ASVs, and 542 ASVs were common to both groups (Figure 1A). The rarefaction curve approached a plateau with increasing sequencing depth, indicating that the sequencing effort was sufficient for the analysis (Figure 1B).

### 3.4 The diversity of the rumen microbiota in HH and TNH sheep

**Figure 2:**
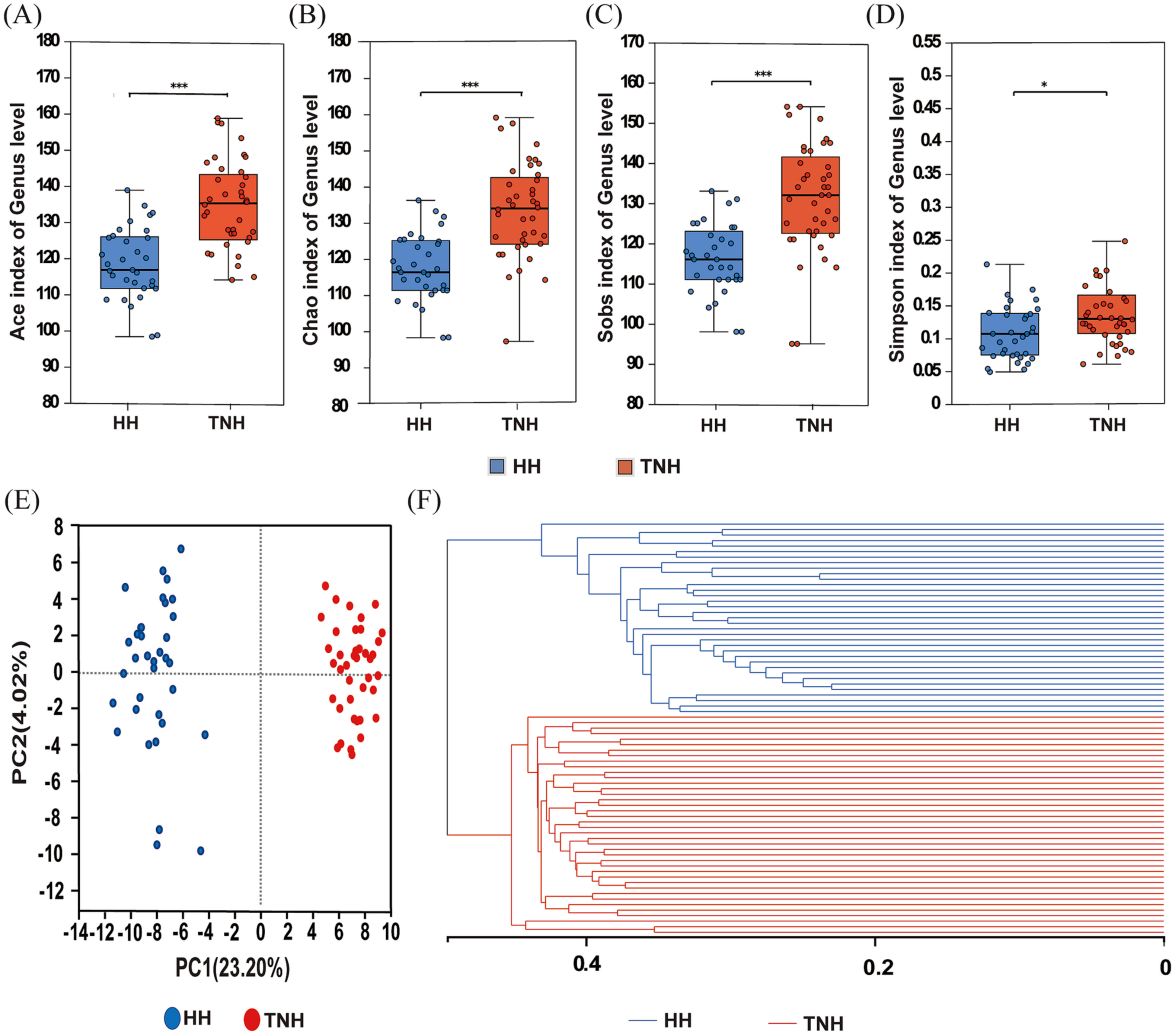
Microbial community structure and diversity analysis in TNH and HH sheep. **(A)** Ace index. **(B)** Chao index. **(C)** Sobs index. **(D)** Simpson index. **(E)** The principal component analysis (PCoA) on genus level. **(F)** The hierarchical clustering on ASV level. The significant difference is indicated by *p* < 0.05.

Alpha diversity analysis revealed that Sobs, ACE, Chao, and Simpson indices in the TNH group were significantly higher than those in the HH sheep (*p* < 0.05) (Figures 2A–D). PCoA based on Bray-Curtis distance at the genus level revealed differences in microbiota composition between HH and TNH sheep (Figure 2E). PCoA1 and PCoA2 explained 23.20 and 4.02% of the total variation, respectively (*R* = 0.5088, *p* = 0.001). Hierarchical cluster analysis of rumen microbiota composition based on Bray-Curtis distance revealed significant differences between TNH and HH sheep (Figure 2F).

### 3.5 Rumen microbiota composition in HH and TNH sheep

**Figure 3:**
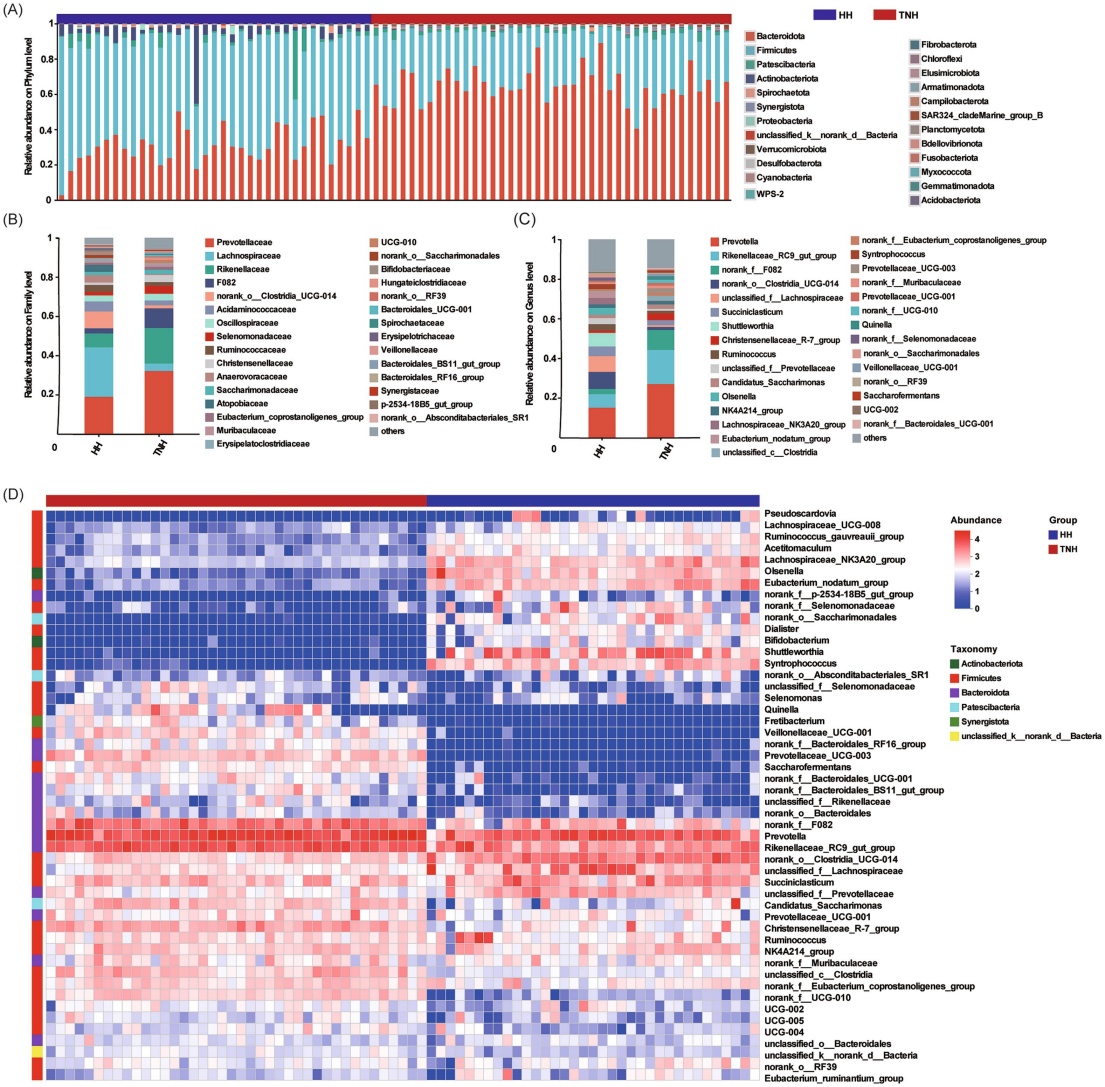
Features analysis of the microbial community in TNH and HH sheep. **(A)** Community barplot at the phylum level. **(B)** Community barplot at the family level. **(C)** Community barplot at the genus level. **(D)** Heatmap analysis of the microbial composition (Top≥50, log_10_). The horizontal axis represents the sample name, and the vertical axis represents the species name.

At the phylum level, the five most abundant phyla in TNH vs. HH sheep were Firmicutes (30.21 ± 1.36% vs. 58.68 ± 1.88%), Bacteroidota (64.75 ± 1.56% vs. 30.99 ± 1.79%), Patescibacteria (2.36 ± 0.21% vs. 3.60 ± 1.15%), Actinobacteriota (0.20 ± 0.02% vs. 4.99 ± 1.20%), and Spirochaetota (0.43 ± 0.06% vs. 0.49 ± 0.13%). However, no significant differences were observed between the two groups for any of these phyla (Figure 3A). At the family level, the dominant bacteria in the TNH group were Prevotellaceae, Rikenellaceae, F082, Lachnospiraceae, Acidaminococcaceae, Oscillospiraceae, Selenomonadaceae, Christensenellaceae, Saccharimonadaceae, and unclassified_c_Clostridia. The dominant bacteria in the HH group were Lachnospiraceae, Prevotellaceae, Rikenellaceae, F082, norank_o_Clostridia_UCG_014, Acidaminococcaceae, Oscillospiraceae, Ruminococcaceae, Anaerovoracaceae, and Atopobiaceae. Notably, Atopobiaceae was detected exclusively in the HH group (Figure 3B). At the genus level, the dominant bacterial genera in the TNH group were *Prevotella*, *Rikenellaceae_RC9_gut_group*, *norank_f_F082*, *Succiniclasticum*, *Christensenellaceae_R7_group*, *unclassified_c_Clostridia*, *Prevotellaceae_UCG_003,* and *Candidatus_Saccharimonas*. The dominant genera in the HH group were *Prevotella*, *norank_o_Clostridia_UCG_014*, *unclassified_f_Lachnospiraceae*, *Rikenellaceae_RC9_gut_group*, *Shuttleworthia*, *Succiniclasticum*, *Olsenella*, *Ruminococcus*, *Lachnospiraceae_NK3A20_group*, *Eubacterium_nodatum_group*, *norank_f_F082,* and *Syntrophococcus*. The composition of dominant genera differed significantly between the two groups. In contrast, *Shuttleworthia*, *Olsenella,* and *Syntrophococcus* were detected exclusively in the HH group, while *Prevotellaceae_UCG_003* was presented in the TNH group (Figure 3C). Furthermore, abundance-based clustering analysis revealed that samples from the TNH and HH groups clustered separately, with the abundance of *Prevotella* (phylum Bacteroidota) being a major distinguishing factor (Figure 3D).

**Figure 4:**
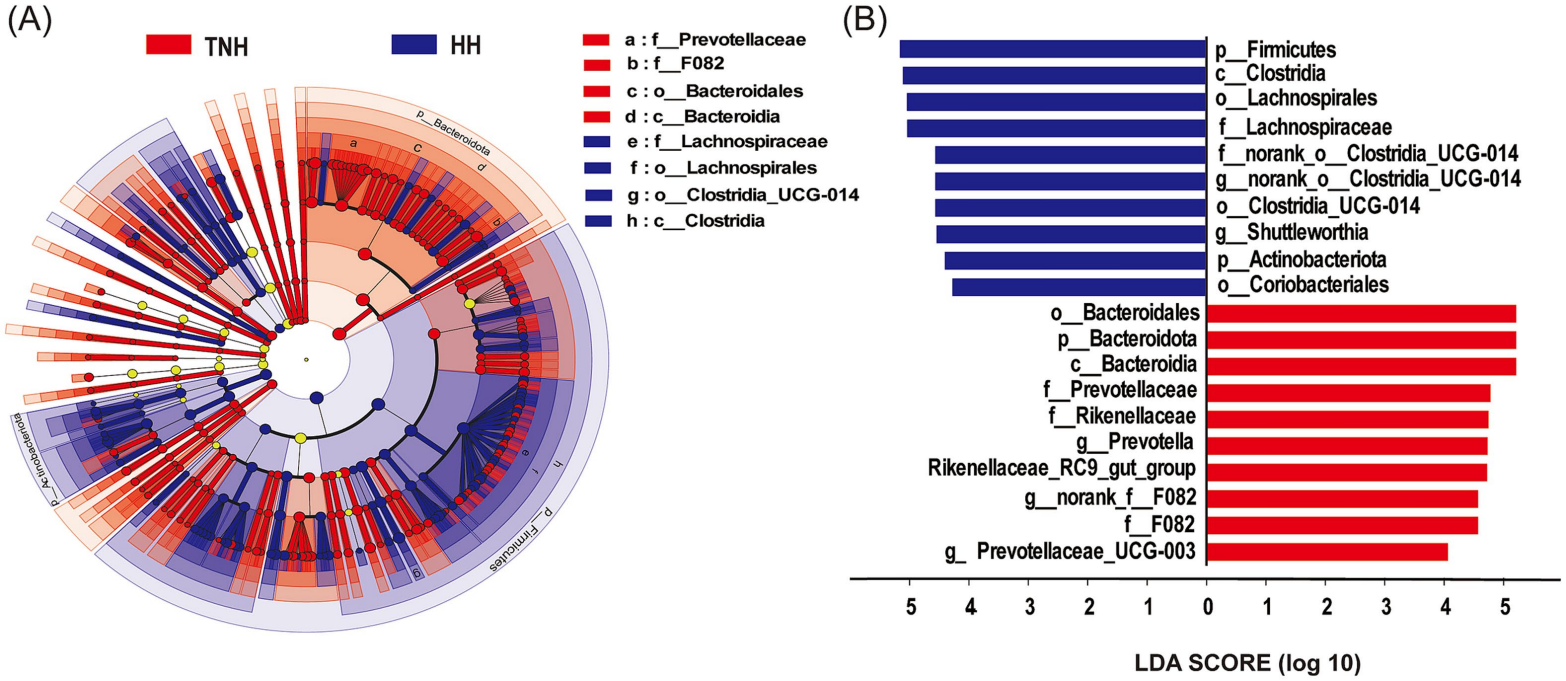
Analyze the significant difference biomarkers between HH and TNH sheep based on LEfSe. **(A)** Evolutionary branch diagram. **(B)** Linear discriminant analysis (LDA > 4).

The differentially abundant bacteria from phylum to genus level between the HH and TNH groups were identified by LEfSe analysis (LDA > 4). A total of 3 phyla, 2 classes, 4 orders, 5 families, and 6 genera were identified as being significantly different. At the phylum level, the HH group had significantly higher abundances of Firmicutes and Actinobacteriota, while the TNH group had significantly higher abundance of Bacteroidota. At the class level, Clostridia was significantly higher in the HH group, while Bacteroidia was significantly higher in the TNH group. At the family level, the abundances of *Lachnospiraceae* and *norank_o_Clostridia_UCG_014* were significantly higher in the HH group, while the abundances of Prevotellaceae, Rikenellaceae, and F082 were significantly higher in the TNH group. At the genus level, the relative abundances of *Prevotella*, *Rikenellaceae_RC9_gut_group*, *norank_f__F082,* and *Prevotellaceae_UCG-003* were significantly higher in the TNH group, while *norank_o__Clostridia_UCG-014* and *Shuttleworthia* were identified as biomarkers in the HH group (Figures 4A,B).

### 3.6 Functional prediction of rumen microbial in HH and TNH sheep

Subsequently, the PICRUSt2 software was employed to predict the KEGG functions of the microbiota in the HH and TNH groups (Table 5). At the first level, metabolism was the main enrichment pathway, accounting for 84.70 and 86.94% in the HH and TNH groups, respectively, and included global and overview maps, carbohydrate metabolism, amino acid metabolism, and nucleotide metabolism. Then, the top 20 functional pathways at the level 3 were compared between the two groups. Pathways related to metabolism, such as Metabolic pathways, Biosynthesis of secondary metabolites, Microbial metabolism in diverse environments, Carbon metabolism, Alanine, aspartate and glutamate metabolism, Amino sugar and nucleotide sugar metabolism, Glycolysis/Gluconeogenesis, Purine metabolism, and Pyrimidine metabolism, were significantly higher in the TNH group than in the HH group. In contrast, pathways including Biosynthesis of amino acids, 2-Oxocarboxylic acid metabolism, Cysteine and methionine metabolism, Starch and sucrose metabolism, Aminoacyl-tRNA biosynthesis, ABC transporters, Two-component system, Quorum sensing, and Flagellar assembly were higher in the HH group than in the TNH group.

**Table 5:** KEGG functional prediction analysis between the HH and TNH sheep.

Level 1	Level 2	Level 3	HH, %	TNH, %	*p-*value
Metabolism	Global and overview maps	Metabolic pathways	31.54 ± 0.46	33.16 ± 0.43	<0.001
		Biosynthesis of secondary metabolites	15.97 ± 0.26	16.41 ± 0.17	<0.001
		Biosynthesis of amino acids	7.68 ± 0.20	7.22 ± 0.15	<0.001
		Microbial metabolism in diverse environments	7.31 ± 0.16	7.46 ± 0.10	<0.001
		Carbon metabolism	4.85 ± 0.11	5.07 ± 0.07	<0.001
		2-Oxocarboxylic acid metabolism	1.51 ± 0.08	1.46 ± 0.07	0.006
	Amino acid metabolism	Cysteine and methionine metabolism	1.94 ± 0.05	1.88 ± 0.03	<0.001
		Alanine, aspartate and glutamate metabolism	1.66 ± 0.03	1.76 ± 0.07	<0.001
	Carbohydrate metabolism	Amino sugar and nucleotide sugar metabolism	1.95 ± 0.09	2.08 ± 0.09	<0.001
		Starch and sucrose metabolism	1.68 ± 0.17	1.46 ± 0.10	<0.001
		Glycolysis/Gluconeogenesis	1.92 ± 0.06	2.03 ± 0.06	<0.001
		Pyruvate metabolism	1.69 ± 0.07	1.69 ± 0.05	0.627
	Nucleotide metabolism	Purine metabolism	2.79 ± 0.07	2.89 ± 0.06	<0.001
		Pyrimidine metabolism	2.17 ± 0.08	2.37 ± 0.10	<0.001
Genetic information processing	Translation	Ribosome	4.52 ± 0.13	4.53 ± 0.09	0.669
		Aminoacyl-tRNA biosynthesis	2.03 ± 0.05	1.98 ± 0.05	<0.001
Environmental information processin	Membrane transport	ABC transporters	3.22 ± 0.31	2.18 ± 0.26	<0.001
	Signal transduction	Two-component system	2.41 ± 0.26	2.03 ± 0.17	<0.001
Cellular processes	Cellular community—prokaryotes	Quorum sensing	2.08 ± 0.12	1.77 ± 0.14	<0.001
	Cell motility	Flagellar assembly	1.05 ± 0.26	0.58 ± 0.15	<0.001

## 4 Discussion

Recognizing the critical role of rumen microbiota in ruminant physiology and nutrient metabolism, this study employed gas chromatography and 16S rRNA sequencing to compare growth performance, rumen volatile fatty acid profiles, and bacterial community composition between HH and TNH sheep raised under identical nutritional and management conditions.

### 4.1 Effect of crossbreeding on growth indicators in offspring

Crossbreeding utilizes heterosis, allowing offspring to inherit superior parental traits and achieve enhanced growth performance. Stature is a highly heritable trait in animals, influenced by numerous small-effect polymorphisms (Bouwman et al., 2018). Consequently, body weight and size are commonly used as key metrics in animal breeding programs. This is particularly true for body weight, a primary economic trait in sheep production. Both body weight and rumen microecology are shaped by genetic and environmental factors, as well as their interaction (Wang et al., 2024; Pang, 2024; Zhang et al., 2026). In the present study, TNH sheep exhibited significantly superior growth performance compared to HH sheep, including higher birth weight, greater average daily gain at various stages, and greater body weight, chest circumference, and tube circumference at 140 days of age. This superiority may be attributed to the higher birth weight of TNH sheep. These findings are consistent with prior reports on crossbred animals. For instance, hybrids such as Dorper × Hu sheep (Gao et al., 2024) and Bobei goats (Zhou et al., 2023) have demonstrated significantly greater body weight, average daily gain, and linear body measurements compared to their purebred counterparts. This alignment across studies robustly supports the developmental advantages conferred by crossbreeding.

Correlation analysis seeks to quantify and interpret the strength of the linear or nonlinear relationship between two continuous variables, thereby uncovering the intrinsic connections among them (Li et al., 2023; Chen et al., 2020). We conducted a correlation analysis of all measured growth indicators. The growth indicators showed varying degrees of correlation with each other. Furthermore, the relationship between body weight and body size measurements exhibited a similar pattern in both HH and TNH sheep. While ADG_0-140 d_ was extremely significantly correlated with multiple traits (BW, BH, BL, and CC) in HH sheep, this correlation in TNH sheep was limited to BW alone. This demonstrates that the development of these indicators is interconnected, collectively influencing the overall growth of an individual. Furthermore, the results suggest that at 140 days, weight gain in HH sheep likely still stems primarily from skeletal growth, whereas in TNH sheep, it arises mainly from muscle and fat deposition. The insignificance of ADG and CC in TNH sheep may imply that muscle and fat deposition are more concentrated on specific areas (such as the limbs) rather than uniformly reflected in the chest circumference. It could be inferred that TNH sheep grow and develop faster than HH sheep. In commercial production, the fattening period for TNH sheep can be shortened, allowing for earlier market readiness, reduced breeding costs, and increased profitability.

The Kleiber ratio (KR) reflects the efficiency of energy metabolism by relating live weight gain to metabolic body weight. Unlike average daily gain, the KR more accurately predicts feed conversion rate as it accounts for the metabolic body weight of the animal, thus serving as a superior selection index for growth efficiency (Vyas et al., 2023). Animals with a high KR are efficient feed utilizers without adverse effects on carcass traits. Furthermore, a higher KR indicates lower maintenance energy requirements, thereby making more energy available for body growth. A significantly higher KR was observed in TNH sheep compared to the HH sheep. This indicates that TNH sheep utilized energy more efficiently for growth and development during the trial. The data demonstrate that the TNH crossbred sheep have inherited a superior meat-yielding body conformation from their Poll Dorset and Southdown sires. Under the same dietary regimen, these introduced advantages in growth rate and meat production have effectively compensated for the inherent limitations of the Hu sheep, as evidenced in their hybrid offspring. Thus, TNH sheep are ideal for producing market-ready fattened lambs.

### 4.2 Effect of crossbreeding on rumen fermentation parameters in offspring

Given that rumen microbial fermentation of feed into volatile fatty acids (VFAs) supplies up to 70% of the host’s energy (Wang et al., 2025; Ge et al., 2023), we analyzed rumen fermentation parameters in lambs to determine the effects of crossbreeding. Our results demonstrate that crossbreeding significantly alters these parameters in the offspring. Specifically, TNH sheep produced significantly higher total rumen VFAs than HH sheep under the same diet. Furthermore, TNH sheep were characterized by an acetate-type fermentation, whereas the HH sheep exhibited a propionate-type fermentation pattern. The acetate-type fermentation serves as the core physiological link underpinning the dual advantages of efficient roughage utilization and superior meat production in TNH sheep. In contrast, the propionate-type fermentation observed in HH sheep channels more energy through gluconeogenesis, resulting in typically lower efficiency and a potential for body fat deposition compared to acetate-type animals. The differentiation in fermentation types at 140 days likely reflects a shift in metabolic priority, where TNH sheep enters a fat-deposition phase, in contrast to HH, which remains focused on rapid weight gain and skeletal growth. Additionally, butyric acid is the most critical regulator and energy source for the normal development, differentiation, and maturation of rumen epithelial cells (Luo et al., 2019). In the digestive system, hybrid vigor is manifested in a more efficient and active rumen microbial ecosystem (Wang et al., 2024). The results of this study partially support that TNH sheep have lower maintenance energy requirements, and crossbreeding could improve growth rate and ruminal fermentation by increasing feed intake and regulating the rumen microbial community and metabolites. In summary, hybrid sheep exhibit advantages in overall energy acquisition and metabolism, and the high yield of rumen VFAs is a core manifestation of an efficient energy acquisition pathway.

### 4.3 Effect of crossbreeding on the composition and function of the rumen microbial community in offspring

Our results showed that TNH sheep have higher species richness and diversity in their rumen microbial community. Previous studies have demonstrated that a more diverse rumen ecosystem contributes to maintaining rumen homeostasis and enhanced resilience to environmental changes (Wang et al., 2024; Konopka, 2009). Functioning as a microbial bioreactor, the rumen’s internal environment is directly regulated by the host’s genome (Wang et al., 2024). Moreover, the hybrid sheep genome, which combines the strengths of its parental genomes, remodels the rumen micro-ecological environment via alterations in physical barriers, immune selection, and metabolic interaction networks (Wang et al., 2024; Du et al., 2025). Consequently, it creates a more stable and diversified micro-habitat for microorganisms. This enables the colonization of microorganisms typically suppressed by or maladapted to the purebred immune system, while simultaneously promoting tighter synergistic interactions among species, ultimately leading to enhanced microbial diversity (Du et al., 2025). Thus, crossbreeding increased the diversity of the rumen microbial community, suggesting an adaptive advantage in fluctuating environments. Similar results have also been obtained in cattle (Zhu et al., 2021), deer (Zhao et al., 2017), goats (Gurelli et al., 2016), and sheep (Cheng et al., 2022).

The results of this study indicate significant differences in the structure and relative proportions of rumen microbial communities between HH and TNH sheep. This finding aligns with Weimer (Weimer, 2015) in that rumen bacterial communities are individual- and host-specific, thus offering a plausible explanation for the differences observed here. We observed that the rumen microbiota of both HH and TNH sheep was dominated by Bacteroidetes and Firmicutes, a pattern consistent with findings in other ruminants including sheep (Wang et al., 2022; Cheng et al., 2022; Lin et al., 2023), goats (Guo et al., 2022), cattle (Gao et al., 2022; Wang et al., 2022), dairy cows (Mu et al., 2022; Zheng et al., 2022), and yaks (Fan et al., 2021). In TNH and HH sheep, Bacteroidetes accounted for 64.75 and 30.99%, while Firmicutes represented 30.21 and 58.68%, respectively. This reciprocal abundance pattern is noteworthy because Wang et al. (Chai, 2024) reported that shifts in the Firmicutes-to-Bacteroidetes ratio-specifically, a decrease in Firmicutes or an increase in Bacteroidetes—are associated with elevated total ruminal volatile fatty acid (VFA) concentrations. These findings confirmed that the total VFA concentration was significantly higher in TNH sheep than in the HH sheep. The divergent relative abundances of Firmicutes and Bacteroidetes between the breeds likely underlie these differences, suggesting distinct rumen fermentation patterns, functional outputs, and efficiencies. Bacteroidetes are primarily involved in the catabolism of readily fermentable carbohydrates (e.g., starch) and proteins, whereas the Firmicutes specialize in the degradation of complex carbohydrates, such as cellulose (Flint et al., 2008).

At the genus level, *Prevotella* occupied a dominant niche within the rumen microbial community of both HH and TNH sheep. Zhu et al. (2021) reported that increasing the relative abundance of *Prevotella* in the rumen effectively promotes lamb growth, a finding consistent with our results. This further confirmed the well-established consensus from previous research that *prevotella* serves as the predominant genus in the rumen of sheep (Wang et al., 2022; Cheng et al., 2022; Li et al., 2024). Its high abundance is indicative of a healthy microbial community and is central to carbohydrate and hydrogen metabolism. While dominant bacteria are crucial for physiological functions, low-abundance bacteria are even more noteworthy. Despite their lower relative abundance, they exhibit greater taxonomic richness and diversity than high-abundance bacteria within the host (Han et al., 2022). For example, *Syntrophococcus.* In addition, these microorganisms establish a reciprocal relationship with the host, influencing diverse phenotypes such as immune activation and pathogen defense. Within their ecological niche, they also interact with other commensal bacteria (Han and Vaishnava, 2023). Significantly, in our study, *Shuttleworthia*, *Olsenella*, and *Syntrophococcus* were detected exclusively in HH sheep. These genera are functional bacteria in the rumen that ferment carbohydrates and produce short-chain fatty acids. Specifically, *Shuttleworthia* encodes sugar ABC transporters, suggesting a specific mechanism for carbohydrate uptake; while the unique syntrophic metabolism of *Syntrophococcus* relies on an intact electron acceptor network within the rumen (Tingga et al., 2026; Krumholz and Bryant, 1986). Such syntrophic communities, characterized by acetate and lactate production, are more likely to colonize the rumen environment of HH sheep. This is not contradictory to the “propionate-type fermentation” phenotype observed in HH sheep but rather reveals the complementarity of rumen microbial functions—HH sheep may possess a complex metabolic network composed of multiple functional bacterial groups (e.g., *Shuttleworthia* contributing to acetate/butyrate production). In contrast, *Prevotellaceae_UCG_003*, a beneficial genus closely linked to fiber degradation (Xu et al., 2025), was enriched exclusively in TNH sheep. This bacterium helps the host acquire energy more efficiently from roughage. This finding is highly consistent with the core phenotypic data of this study, namely that TNH sheep exhibited higher total VFA production and a higher Kleiber ratio, indicating a rumen micro-ecosystem more conducive to fiber degradation and efficient energy utilization in their offspring. In summary, the differences in rumen microbial composition between HH and TNH sheep reflect the selective shaping of the rumen microecology by the host genetic background (purebred vs. crossbred). Crossbreeding, by remodeling the rumen microbial interaction network and metabolic pathways, constructs a more efficient “fiber degradation-energy conversion” axis, thereby conferring significant production performance advantages to TNH sheep.

Microbiota function prediction revealed that metabolic pathways, carbon metabolism, and glycolysis/gluconeogenesis were predominantly enriched in TNH sheep, suggesting a role in regulating carbon partitioning and sugar synthesis/conversion. This functional profile aligns with the predominance of Bacteroidota and Firmicutes, phyla known for their role in digesting complex carbohydrates in the ruminant rumen. In contrast, the relative abundance of pathways related to biosynthesis of amino acids and ABC transporters was higher in the HH sheep. These pathways are closely linked to propionic acid fermentation, whose major end products, propionic acid and acetic acid, function as critical carbon-skeleton precursors for synthesizing amino acids such as alanine and members of the glycine/aspartate family. Crucially, because accumulation of these acids is toxic, cells must efficiently export them—a function that frequently relies on ABC transporters. Collectively, rumen microbes share core metabolic functions, generating energy via microbial metabolism to support host growth. Currently, these functional enrichments are predictive, and the specific pathways require further investigation. Furthermore, we recognize that measurement of blood parameters, NH_3_-N, and enzyme activities would provide additional insights into the systemic metabolic state of the animals and could help explain the observed differences in growth performance. Future studies should incorporate such analyses to further elucidate the rumen metabolic response.

## 5 Conclusion

In summary, this study demonstrated that crossbreeding improved growth performance in sheep by modulating the rumen microbiota. Compared to HH sheep, TNH lambs exhibited superior growth rates and meat production potential. This was accompanied by greater rumen microbial richness and diversity, and a distinct shift toward an acetate-type fermentation pattern. Although both groups shared a core microbiota dominated by Bacteroidota and Firmicutes, TNH sheep harbored a distinct community structure with *Prevotella* as the predominant genus. Notably, *Shuttleworthia*, *Olsenella*, and *Syntrophococcus* were unique to HH sheep, whereas *Prevotellaceae_UCG_003* was specifically enriched in the TNH group. Functional predictions further revealed that pathways related to carbon metabolism and glycolysis/gluconeogenesis were enriched in TNH sheep, while those for biosynthesis of amino acids and ABC transporters were more abundant in HH sheep. These findings suggest that crossbreeding establishes a more efficient “fiber degradation–energy conversion” axis by remodeling the rumen microbial interaction network and metabolic pathways, thereby conferring significant production advantages to TNH sheep. From a practical perspective, this study supports the development of breed-specific feeding strategies tailored to distinct rumen fermentation patterns and microbial profiles. Such precision nutrition approaches could shorten the fattening period, reduce costs, and ultimately improve profitability.

## Data Availability

The raw sequencing data generated in this study are available in the NCBI (https://www.ncbi.nlm.nih.gov/) under accession number PRJNA744748.
